# The Art of Holding Perinatal Women in Distress

**DOI:** 10.1089/whr.2022.0083

**Published:** 2023-03-02

**Authors:** Karen Kleiman, Hilary Waller

**Affiliations:** The Postpartum Stress Center, Rosemont, Pennsylvania, USA.

**Keywords:** postpartum, perinatal distress, supportive psychotherapy, perinatal mental health, therapeutic alliance, postpartum depression, holding environment

## Abstract

Therapies like cognitive behavioral therapy and interpersonal psychotherapy are regarded as highly effective treatments for perinatal mood and anxiety disorders. Therapists appreciate robust research supporting the efficacy of these evidenced-based treatments and the structure of the tools these therapies provide for effective intervention. Less has been written on supportive psychotherapeutic techniques and few of those writings provide instruction or tools for therapists who wish to strengthen their skills in this therapeutic approach. This article describes The Art of Holding Perinatal Women in Distress™, a model of perinatal treatment developed by Karen Kleiman, MSW, LCSW. Kleiman instructs therapists to incorporate six “Holding Points” into their approach to therapeutic assessment and intervention for the purpose of establishing a holding environment conducive to the release of *authentic suffering*. This article reviews the Holding Points and provides a case study that elucidates how the holding points function within the context of a therapy session.

Perinatal depression affects 10%–20% of women in the United States during pregnancy and the postpartum period.^1^ Prevalence estimates are higher among women from African American and Hispanic communities.^2^ Increasing empirical evidence endorses the efficacy of psychological treatments such as cognitive behavioral therapy (CBT)^3^ and interpersonal psychotherapy (IPT),^4^ yet retention in therapy for women suffering from perinatal distress in the United States proves to be poor, with many women choosing not to complete treatment to the point of symptom relief.^5^ It is further noteworthy that despite ongoing medical and educational advances, legislative progress, an increase in public awareness, and access to screening, perinatal women in distress continue to be hindered by overwhelming and enduring barriers. Stigma, shame, and fear of judgment are among the well-known reasons perinatal women refrain from seeking help.^5^

In addition, sociocultural expectations, inadequate resources, financial and logistical barriers such as access to care, and symptoms that interfere with help-seeking behaviors, serve to reinforce a woman's reluctance to disclose the extent to which she is suffering. With this abundance of barriers, perinatal women sit precariously alone with their symptoms and historically exhibit low rates of help-seeking behaviors.^6,7^ Given that suicide accounts for 20% of all postpartum deaths,^8^ stakes for successful therapeutic outcome are high and swift and judicious intervention is essential.

While CBT and IPT are unquestionably effective treatments to achieve relief from symptoms of mood and anxiety disorders in the perinatal period, the efficacy of these interventions is dependent on client engagement in treatment and disclosure of distress. It is well known that the therapeutic alliance is a key component of successful treatment and has been shown to be mild to moderately predictive of a successful treatment outcome.^9^ The therapeutic alliance is further associated with increased client follow through and retention,^10^ While many educational and professional training opportunities now exist for providers wishing to treat perinatal mental health disorders, few focus on ways to develop therapeutic rapport with this nuanced population.

The Art of Holding Perinatal Women in Distress™ was initially presented in Karen Kleiman's 2017 book, *The Art of Holding in Therapy: An essential intervention for postpartum depression and anxiety*.^11^ The Art of Holding Perinatal Women in Distress, or the holding approach, is an intervention developed in response to the recognition that despite clinically meaningful and highly disruptive symptoms of mood and anxiety disorders, many perinatal patients who are symptomatic of perinatal mood and anxiety disorders do not receive treatment. Rooted in observational evidence collected over three decades of clinical practice and research on the therapeutic relationship, Kleiman's holding approach aims to support the swift development of the therapeutic alliance in a patient population that is especially guarded.

The barriers that prevent perinatal women from seeking treatment and following up are impressive and unfortunately, prolong suffering and delay recovery. As more therapists specialize in this unique field of study, it is essential that they are trained in strategies that increase the likelihood that the perinatal woman in distress will feel cared for, and ultimately, engage in the process on some level. Training in The Art of Holding Perinatal Women in Distress provides practitioners insight into the complexity of perinatal psychology and skill building to help clients find support in a therapeutic relationship and worth in pursuing the therapeutic process.

This article introduces the main tenets of the holding approach, including key aspects of the theoretical framework and discussion of the six holding points, skills that guide the clinician's response to a perinatal client's expression of distress. A case example is included to illustrate how consideration of the holding points impacts the clinician's thought process, assessment of client presentation, and efforts to engage the client in therapy.

## The Holding Environment

Introduced by D.W. Winnicott, the holding environment was conceptualized as fundamental to infant development of self-regulation skills, an individuated sense of self, and the establishment of trust and safety in relation to others.^12,13^ Winnicott applies this concept to the therapeutic alliance, comparing the therapeutic space to a holding environment intended to facilitate the development of client self-regulation, individuation, trust, and safety.^14^ Like the infant's holding environment, at its most basic level, the therapeutic holding environment similarly establishes a consistent, well-boundaried, reliable, and empathic setting in which the client can thrive and begin to heal. The ultimate experience of having her primary needs met *via* feeling *held* while emotionally falling apart enables the client to trust the environment, relinquish control over her pain and fear, and feel better equipped to resume functioning with a stronger sense of agency.

Kleiman's *The Art of Holding in Therapy: An essential intervention for postpartum depression and anxiety*^15^ finds resonance in Winnicott's work and expounds on his analogy. The holding approach provides a framework for establishing a therapeutic holding environment for perinatal women.

Reinforcing Winnicott's recognition that the self of therapist and the therapeutic relationship are primary agents of change and healing,^16^ Kleiman outlines six *holding points* (Table 1), which incorporate concepts from traditional supportive psychotherapy as well as cognitive and mindfulness-based approaches, to establish emotional alignment and guide the therapist's responses to the client and to the therapeutic process. The *holding points*: grounding, current state, expert, design, presence, and safeguarding, may be understood as the ingredients for the skillful development of the therapeutic holding environment. Kleiman suggests that those practitioners who approach a perinatal woman with these skills in mind are best positioned to establish a containing therapeutic environment in which attunement and empathy can emerge.

**Table 1.** Description of the Holding Points as Presented in The Art of Holding Perinatal Women in Distress by Karen Kleiman

• Grounding: The therapist's use of self, including modulation of voice and nonverbal cues to restore balance to a client's activated state. • Current state: The therapist's ability to attend to her symptoms and initiate a rapid evaluation of the client's physical safety, level of distress, and level of functional impairment. • Expert: The therapist's capacity to maintain humility while simultaneously communicating competence and confidence in their ability to carry high levels of distress and implement treatment for the client's symptoms. • Design: The therapist's collaborative blueprint of an initial treatment plan, which centers on the client's stated needs. • Presence: The therapist's capacity to establish and sustain the “holding environment” while simultaneously maintaining self-awareness, relational attunement, and boundaries. • Safeguarding: The therapist's role in balancing the need for protection and support while facilitating client autonomy and the development of human agency. The Holding Points are further differentiated as tools for urgent symptom relief and techniques to facilitate relational healing and transformation: • Urgent symptom relief: Grounding, Expert, Design • Relational healing and transformation: Current state, Presence, Safeguarding

## Authentic Suffering

Kleiman uses the term “authentic suffering” to describe the unique intersectionality of the perinatal adjustment and symptoms of a mood or anxiety disorder. Perinatal distress and resistance to help seeking are often characterized by the beliefs that others may not understand their suffering, may judge their suffering, or may overreact to their suffering. As a result, new mothers begin to develop an unwavering instinct for protection from the outside world. This urge to hide from the terror of the unknown, from their own ego dystonic intrusive thoughts and from the shame that drives their symptoms, is a level of suffering they often do not feel prepared to disclose. Kleiman names this deep, internal, and protected distress, *authentic suffering*.

Kleiman describes authentic suffering as:

That which is obscured by what she wants us to know and what she will let us see.

It is the pain she conceals.

It's the terror that immobilizes her and keeps her up at night.

It's what drives her anxiety and her fear that she will continue to descend, endlessly.

It's what's in her suicide note when loved ones cry out that, *there were no warning signs*.^17^

Kleiman finds that authentic suffering is often embedded in a perinatal woman's steadfast wish to cling to one or many aspects of her previous self, which she feels has been compromised by the transition to motherhood. Identification with this suffering, as a reflection of her former self, creates a façade of comfort and a longing for privacy. Even supportive gestures and interventions may be perceived as intrusions and threats to this part of herself, creating a paradoxical opposition to anything that might bring relief. Therapists recognize this inertia as resistance.

Resistance may come in the form of opposing therapy. Or it may be in her reluctance to disclose the extent to which she is suffering. Or, it may be her deep-seated belief that getting help will make things worse. In desperation, she clings to her *self*, in an attempt to hold on to her very being. The notion of letting go of this resistance is fundamentally disconcerting and provokes a primitive agony—that she will experience “unthinkable anxiety” resulting in her “falling forever.”^18^ When clinicians fail to access the authentic suffering of a perinatal woman, they merely scratch the surface of despair, a despair which can veil the potential of her transforming self and reinforce her catastrophic thoughts or inclinations.

## Case Report: Rachel

Rachel, a 32-year-old Caucasian mother of a 2-month-old baby girl, called The Postpartum Stress Center after experiencing “weeks of crying and just not feeling like myself.” She reported a family history of depression and personal experience with depression in college. No previous psychiatric medication or hospitalization. She reported a stable relationship with her partner, to whom she has been married 4 years. She works as a cardiac nurse in a local hospital. Rachel loves her job but expresses ambivalence about returning after maternity leave. She expresses frustration at feeling so “incompetent and utterly useless,” leaving her to wonder why she had a baby in the first place.

Upon being greeted, Rachel apologized as she handed back the forms that were left for her in the waiting room. “I didn't have time to fill these out. Sorry.”

“That's okay. We can go through them together. Let's just talk.”

Rachel sat forward on the edge of the couch, not quite settling into the moment.

“This didn't work with the other therapist I saw,” she smirked.

“What didn't work?”

“This. I went to see this other therapist and I'm not sure why exactly, but it wasn't helpful. She basically said I was fine.”

“And?”

“I'm not fine.”

“Why do you think she said you were fine?”

“I dunno. Cause I *look* fine? Cause I answered her questions the way she thought I should?”

We sat quietly.

“Was there more that you wanted to say to her?” I asked.

Rachel tilted her head and shrugged, “Maybe. She was asking questions that sounded like she was reading off a list in her head. I was afraid she was labeling me or something. She wasn't really getting it.”

“She wasn't getting it? Can you describe to me what you mean when you say that?”

Rachel softly inhaled.

“She wasn't getting how I'm feeling. She didn't do anything wrong. I just felt like I was her 2 o'clock appointment with a problem she had to solve. I just felt awkward sitting there.”

I leaned in and listened. Mostly, I looked, my eyes fixed on hers. I breathed deeply and slowly as I absorbed her distress signals.

“I can only imagine how it feels when you gather the courage to come to an appointment like this when you have just had a baby and are unsure about how you feel. It's not an easy thing to do. It's hard to talk to a stranger about emotions, especially when you are vulnerable and overwhelmed.”

“Yeah, it's hard.”

“Let's sit, okay? Let's put the paperwork down and just sit for a minute, together.”

“Okay. Thanks.” Rachel took another breath.

“Good. Let's just sit. And breathe.”

A brief moment passed.

“You okay right now?”

“That was nice.” Her face brightened a bit.

“What was nice?”

“I dunno. Just asking me to sit, I guess, and be quiet for a minute so I could take a breath. And asking me if I'm okay.”

I nodded.

“I really don't feel good.”

“I know.” I spoke gently, maintaining eye contact and breathing in sync with her. I rested in place and waited.

“I don't know what's happening to me but everything feels wrong. My brain. My heart. Everything hurts.

We sit.

“I'm so tired.” Rachel lowered her head with closed eyes.

I wait.

“It's good you're here, Rachel. I know it isn't easy to be here.”

She takes a deep breath in, as if to absorb the reassurance.

“Tired can mean so many things to new mothers. Can you tell me what tired feels like to you?”

Quietly Rachel spoke, looking down and her hands, she appeared to shrink into the couch. “I'm tired. I am sleep deprived from caring for my baby, it feels like we're up all night. But I feel more than just tired, I feel exhausted in every single way. And honestly it isn't just her waking me. When she is sleeping, I can't sleep because I am worrying that I will always feel like this. I'm thinking about whether my husband understands, whether he will ever understand. I feel so lonely in the middle of the night, but then when I look forward to morning, I remember I will be alone again for most of the next day caring for my daughter. I love her but I'm afraid I never should have had a baby. I'm afraid I'll be a terrible mother. I AM a terrible mother so far. I'm afraid I will never become a good mother. I feel trapped and I'm worried will never ever be okay again. I'm just so tired,”

“These are really heavy thoughts to carry. No wonder you're tired.”

As the session progressed, Rachel elaborated on how her depressive symptoms and pervasive exhaustion manifested. All of what ensued in that initial session was unsurprising as far as the course of perinatal assessments go.

## Discussion

A brief review of this session excerpt will serve to elucidate the use of holding points. Upon entering the office, Rachel first apologizes then sits on the edge of the couch. In this moment the client's current state is observed. Noticing her restlessness, Kleiman reassures her that they can talk through the paperwork together and prioritizes building rapport, stating, *let's just talk.* Kleiman observes that Rachel's agitation is quite conspicuous, suggesting that grounding would help her feel cared for, so attention is paid to voice regulation, slowing down, and softening the tone to project a calming energy. Eye contact is unflinching yet sincere. Focus is steady, sensitive to the unpredictable nature of distress, and it is directed squarely onto the client. This quiet attention appeared to soothe Rachel and she began to interact soon after sitting down.

Once Rachel begins speaking, she describes feeling discouraged, stating, *This didn't work with the last therapist I saw*. It is notable that she comes to therapy with low expectations, which can influence clients' readiness for change and perception of the therapeutic process.^19^ Paradoxically, this negativity may also reflect access to her authentic suffering. This comment causes Kleiman to wonder whether Rachel is experiencing hopelessness related to the therapeutic process. Curious about whether hopelessness could be impacting Rachel's current state, Kleiman decides to insert early stages of hope by conveying confidence in her ability to help Rachel obtain symptom relief. The concept of expert can be challenging for therapists to embody as it can elicit feelings of self-doubt and an overidentification with the imposter syndrome. It is, nonetheless, a crucial posture for the therapist to cultivate and refine, as a failure to do so can impact the therapeutic trajectory as well as the therapeutic relationship.^20^

In this example, we see how the therapist uses a validating statement that correctly intuits an unspoken aspect of the client's distress: “I can only imagine how it feels when you gather the courage to come to an appointment like this when you have just had a baby and are unsure about how you feel. It's not an easy thing to do. It's hard to talk to a stranger about emotions, especially when you are vulnerable and overwhelmed.” Often therapists find it helpful to utilize a normalizing or psycho educational statement to embody the expert voice.

Kleiman then checks in on Rachel's level of distress, asking the question, *are you okay right now?* Free from clinical jargon and expressed with caring, familiar ease, these words are an initial expression of safeguarding. Here the therapist presents as protective and attentive to the client's needs and conveys that the client's feelings are paramount and always embolden the session. If she is okay, we proceed. If she is not okay, we pause to attend to her changing current state. In this case, when asked if she is okay, the client expressed appreciation of this consideration. This comment may be an indication that others have rejected her current state, or she felt unworthy of expressing this, or perhaps there is so much attention to the new baby she felt her needs were less important. Rachel then reveals, *I really don't feel good,* an early hint that there is more to her authentic suffering, and soon thereafter, *I'm so tired,* a deeper acknowledgment of her suffering.

Once any potential for emergency intervention, such as suicidal thoughts, has been denied, the holding response to authentic suffering is to lean in with empathic resolve and sit with the suffering. When therapists are able to successfully *sit with suffering*—that is, skillfully tolerate acute distress by managing to control both the client's discomfort and their own reaction to it—they embody holding. The act of sitting with suffering requires that the therapist simultaneously apply *all* holding points, while monitoring their own somatic and emotional experience.

This conflation of experiences, the client's expression of authentic suffering, the therapist's empathetic and concurrent use of grounding, current state, expert, design, presence, and safeguarding results in a profound shared experience. The exchange of energy and understanding of need between the client and therapist is what Kleiman refers to as therapeutic “magic.” This “magic,” an element of empathy, reinforces safety within the holding environment, enabling release of authentic suffering and the space for transformation.^21^

The words, *I'm tired*, in and of themselves, when uttered by a pregnant or postpartum mother can be easily misunderstood by a caring support person who responds with reassurance that “all moms are tired” and “it will get better soon.” In the context of therapy for perinatal distress it is important to avoid use of such truisms as any symptomatic expression is recognized as a potential clue, a fragment of the pain she conceals. In Rachel's case, *I'm tired* meant that she is, in fact, tired and sleep deprived due to the demands of her infant. Her statement *I'm tired*, however held much deeper meaning. When Kleiman asked Rachel to describe what these words mean to her, Rachel revealed that:

1. She is, in fact, sleepy from receiving inadequate rest/sleep.
2. She is weary from the day-to-day demands of childcare.
3. She is coping with feelings of isolation throughout the night and during the day.
4. She is experiencing intrusive thoughts that are keeping her up at night.
5. She is worried about her partnership and feeling distant from her husband.
6. She is experiencing negative thoughts about herself.
7. She is worried about whether she will be a good mother.
8. She is convinced that she has been a bad mother so far.
9. She feels that she is not okay and that this will continue forever.
10. She feels trapped.

When Kleiman asks Rachel to tell her more about feeling tired, she opens the door for Rachel to disclose her fatigue as the all-encompassing depiction of her despair. In this case, the way she describes it, the whispered voice she used and her contracted body language are consistent with the hopelessness she expressed at the outset of the session.

Using holding points as a reference, and in no particular sequence, Kleiman ensures that Rachel is grounded by waiting, and allowing Rachel to sit quietly to see how it feels for her to disclose this. Kleiman offers reassurance in the form of a grounding statement, “It's good you're here, Rachel. I know it isn't easy to be here.” Here, she safeguards Rachel from her overwhelming feelings and reassures her she is in the right place. In response to perinatal overwhelm, safeguarding entails a delicate balance between normalizing this state without being dismissive or presumptuous. Observing her current state helps determine if it feels comfortable to proceed or if Rachel needs help managing the emotions by way of verbal support, or specific grounding strategies.

Assuming the expert stance, Kleiman interprets and responds to Rachel's tiredness with open-ended questions, which convey to Rachel that she does not assume that *tired* is simply the result of infant-related sleep disturbance. This invitation to explore and express the meaning of *tired* is met with relief since often peers, loved ones, and medical providers dismiss fatigue as a normal and predictable postpartum experience, and this relief enables Rachel to open further. By responding attentively to Rachel's expression of tiredness, Kleiman discards the notion of completing the paperwork and pivots the early clinical protocol toward an intervention that felt more attuned to Rachel's distress. This design to focus on her immediate suffering, contain the distress, and defer administrative drills is a hallmark feature of perinatal clinical practice.

When Rachel voices her concern about always feeling like this and over identifies with being a *terrible mother,* the scope of her authentic suffering becomes clearer. Staying present alongside the strong negative emotions with acceptance and without alarm is another cornerstone of perinatal clinical practice. The capacity to contain extraordinarily high levels of distress and make that look easy and natural is a prerequisite for any maternal mental health therapist. The raw emotions of perinatal women in distress are easily bruised and ripe for misinterpretation by the client. The therapist must stay connected to the expert holding point when exhibiting language, tone, posture, facial expressions, eyebrows, mouth, eye contact, breathing—all of which can inadvertently convey potentially detrimental messages. At the same time, the therapist must intermittently take inventory of their own affective state by blocking any internalization of the suffering and monitoring their emotions.

## Limitations of This Article and Approach

Regardless of their primary therapy modality, mental health care providers specializing in this nuanced population widely agree that integration of supportive psychotherapy is an essential component to the therapeutic alliance with perinatal clients. The relevance and salience of the holding approach will be bolstered by clarifying its utility and identifying best practices for integration when appropriate. This is a topic requiring further study as the holding approach is not intended to replace or exclude other commonly used, evidenced-based psychological interventions such as CBT and interpersonal therapy (IPT).

It is interesting to note that the holding approach has piqued the interest of clinicians with diverse theoretical orientations. Some clinicians trained in CBT report that holding strategies create a framework which deepens their conceptualization of the perinatal distress itself, which, in turn, enriches their therapeutic connection and process. This is one example of how the overlap of CBT protocol and the addition of holding skills can potentially improve the therapeutic relationship, treatment effectiveness, and ultimate outcome.

A significant limiting factor impacting further study of the holding approach is difficulty developing quantitative measures for supportive psychotherapeutic concepts. Operational definitions for these approaches can be elusive and empirical examinations sparse compared with therapies such as CBT or IPT. Clearly, the value and validity of literature focusing on the holding approach will be significantly increased with robust empirical exploration. At this stage in the development of the holding theory, client and therapist anecdotes best illustrate how holding serves to build trust and develop attunement with reluctant perinatal clients. Despite the limitations inherent in reliance on this anecdotal evidence, it behooves psychotherapy providers to consider the utility of integrating aspects of the holding approach to their practices.

## Conclusions

Psychological, nonpharmacological treatments are the choice of treatment for the majority of perinatal women.^22,23^ As illustrated in this case report, the subtleties unique to perinatal distress provide rich clinical material, which may be more accurately understood and interpreted when viewed through a lens specifically attentive to the perinatal stage. Therapists who are trained in holding techniques position themselves to be more attuned to these intricacies. Because this highly vulnerable population of women is likely to postpone seeking or disengage from treatment,^24^ it is incumbent upon the clinician to increase the likelihood of therapeutic connectedness. When depressive and anxious symptoms are significant, resistance to psychotherapy is often strong and challenging for both the mother in distress and the clinician. Training in the holding approach encourages development of the skills needed to ensure that this connection is possible.

## Abbreviations Used

CBT: cognitive behavioral therapy
IPT: interpersonal psychotherapy
